# RASopathies due to de novo pathogenic variants: clinical features, genetic findings and outcomes in nine neonates born with congenital heart defects

**DOI:** 10.1186/s12920-022-01336-3

**Published:** 2022-08-24

**Authors:** Simin Zheng, Huanyang Huang, Li Ma, Tianwen Zhu

**Affiliations:** 1 Department of Neonatology, Xinhua Hospital, Shanghai Jiao Tong University School of Medicine, Shanghai, China; 2 Department of Neonatology, Shanghai Children’s Hospital, Shanghai Jiao Tong University School of Medicine, Shanghai, China

**Keywords:** RASopathies, Neonatal, Congenital heart defect, Family-based exome sequencing, De novo mutation

## Abstract

**Background:**

There are limited information available related to neonatal characteristics of RASopathies, a group of autosomal dominant syndromes with considerable phenotypic overlap.

**Methods:**

The retrospective review revealed 9 neonates born with congenital heart defects (CHDs) and diagnosed as RASopathies due to de novo mutations (DNMs) by trio-based exome sequencing (ES) between January 2017 and December 2020. We report in details of the neonatal course, molecular analysis and 180-days of age follow-up in affected individuals.

**Results:**

The early clinical spectrum included various types of CHDs, less noticeable multiple extracardiac anomalies and unspecific symptoms like poor feeding. Of the 8 variants identified from 6 genes, 2 in *RASA1* were novel: (NM_002890.2: c.2828 T > C (p.Leu943Pro)) and (NM_002890.2: c.2001del (p.Pro668Leufs*10)), which functionally impaired the protein structure. There was a relatively high mortality rate of 33.33% (3/9) for all the defects combined. A *RAF1*-deficient male and a *RASA1*-deficient male survived from severe heart failure by surgical interventions in early life.

**Conclusions:**

Our results revealed that family-based ES was useful in identifying DNMs and causal genes for sporadic diseases and screening Rasopathies shortly after birth. We recommended a family-based ES and a full phenotypic evaluation including echocardiogram, magnetic resonance imaging, ultrasonography and coagulation screening in neonates with CHDs and a suspected genetic etiology.

## Background

CHD accounts for nearly one-third of all congenital birth defects [1] and, therefore, the focus on CHDs is integral to eliminating preventable infant deaths. Recent preclinical [2] and clinical [3] have indicated a genetic etiology for CHDs. Another large genetic study of CHDs with next-generation-sequencing (NGS) [4] suggested that 8% and 2% of cases are attributable to de novo autosomal dominant and inherited autosomal recessive variation, respectively. These studies contributed to the understanding of the genetic etiology of CHDs and the underlying molecular mechanisms. This, in turn, will enable a multidisciplinary care to these individuals to extend lifespan and improve health [5].

The RASopathies are a group of autosomal dominant disorders with overlapping cardiac, facial, and neurodevelopmental features [6]. RASopathies include Noonan syndrome (NS) (MIM 163950), Noonan syndrome with multiple lentigines (NSML) (MIM 151100), Noonan-like syndrome with loose anagen hair (NS-LAH) (MIM 607721), cardio-facio-cutaneous syndrome (CFC) (MIM 115150), Costello syndrome (CS) (MIM 218040), Neurofibromatosis type 1 (MIM 162200), Legius syndrome (MIM 611431) and capillary malformation-arteriovenous malformation (CM-AVM) (MIM 608354) [7]. Taken together, the RASopathies represent one of the most prevalent groups of malformation syndromes affecting greater than 1 in 1,000 individuals equally distributed in females and males [6]. While most individuals with RASopathies share characteristic findings affecting multiple organ systems, the phenotypic spectrum is wide, ranging from a mild or attenuated phenotype to a severe phenotype with infantile lethal complications [8]. Furthermore, less major clinical features were recognized in neonatal or infantile period [9] than the manifestations in toddlerhood and beyond. This non-specificity of presentations means that clinically diagnosing RASopathies can be challenging in early life. Hence, this study aims to describe the neonatal features of RASopathies with de novo causative variants (the most extreme form of rare genetic variations) in order to facilitate prompt clinical diagnosis, enhance patient management and family counseling.

## Methods

### Patients and clinical data

This is a retrospective analysis of clinical records for neonates with various CHDs and a confirmed molecular diagnosis of RASopathies due to de novo pathogenic variants from two tertiary hospitals in China between January 2017 and December 2020. The institutions included Xinhua Hospital affiliated with Shanghai Jiao Tong University School of Medicine (XH) and Children's Hospital affiliated with Shanghai Jiao Tong University (CH). The institutional review board at Xinhua Hospital, Shanghai Jiao Tong University School of Medicine approved this study with a waiver of informed consent due to the nature of the study (Approval number: XHEC-D-2022-077).

All subjects were admitted to the corresponding neonatal intensive care units (NICUs) from two hospitals immediately after birth or shortly after. The sociodemographic, baseline characteristic, and phenotypic data were extracted from electronic medical records (EMRs) and the phenotypes of the affected neonates were further translated into Human Phenotype Ontology (HPO) terms [10]. All patients were followed up till 180 days of age. When a patient died, the investigator was asked to identify the primary causes of death and to assess the relationship of death to the primary disease.

### Exome sequencing and molecular analysis

Those individuals that were performed with trio-based exome sequencing (ES) and analysis were included in our final study. Detailed sequencing and analysis information can be found in our previously published paper [11]. Only detected single-nucleotide variants (SNVs) and copy number variations (CNV) in *RASA1*, *HRAS*, *BRAF1*, *CBL*, *KRAS*, *NRAS*, *PTPN11*, *RAF1*, *RRAS*, *RRAS2*, *RIT1*, *SHOC2*, *SOS1, SOS2*, *MAP2K1 (MEK1)*, *MAP2K2 (MEK2)*, *SPRED1* and *NF1* were further interpreted and categorized according to the guidelines recommended by the American College of Medical Genetics [12, 13] for the corresponding SNV and CNV results. They were also addressed as novel or reported according to entries into the Human Gene Mutation Database (http://www.hgmd.org) [14] and the Genome Aggregation Database (gnomAD; http://gnomad.broadinstitute.org) [15]. The following available online analysis tools were also used to predict the effect of the novel missense variants. Polymorphism phenotyping (PolyPhen-2) [16] was used to predict their pathogenic effects. The multiple-sequence alignments were carried out by T-Coffee Multiple Sequence Alignment Program [17]. The protein structure analysis was performed by Project HOPE [18].

## Results

### Demographic feature

Data collection for this study began January 1, 2017 and ended on December 30, 2020, when 325 neonates with various CHDs had been clinically evaluated and ES tested. Patients with incomplete clinical EMRs or family-based ES data not yet fulfilled were excluded. Totally, 9 patients were identified with a de novo pathogenic or likely pathogenic variant that established a genetic diagnosis of RASopathies. Nine RASopathies were sporadic cases and they were categorized into NS and Noonan-related disorders in six patients (Patient (P)1, P2, P3, P4, P5 and P6), CM-AVM in two (P7 and P8) and CS in one (P9). Our cohort included 6 males and 3 females whose ages ranged from at birth and 23 days after birth (median: 1 day after birth) at the time of admission to NICUs. Two pregnancies (P8 and P9) were complicated by polyhydramnios. Nuchal cystic hygroma and renal anomalieswere prenatally detected in 2 patients (P2 and P3) by prenatal ultrasound. Two patients (P4 and P8) (22.22%, 2/9) were born before 37 weeks of gestation. Additional demographic characteristics of the individuals are summarized in Table 1.

**Table 1:** Clinical and laboratory data of the nine neonates with RASopathies

Patients	Patient 1	Patient 2	Patient 3	Patient 4	Patient 5	Patient 6	Patient 7	Patient 8	Patient 9
Gender	M	M	M	M	M	F	M	F	F
Age at presentation(days)	5	0	23	2	0	6	0	0	1
Gestation Age (weeks)	39.86	38.29	41	36.14	39.29	37	40.43	31.86	37.29
Birth weight (g)	3800	3050	4750	3350	3620	3760	4400	2735	3505
Birth length (cm)	51	48	56	49	48	49	56	46	48
Paternal age (years)	34	43	32	Unknown	28	30	35	37	35
Prenatal assessment	Uneventful	Nuchal cystic hygroma and renal abnormality	Nuchal cystic hygroma and renal abnormality	Uneventful	Uneventful	Uneventful	Uneventful	Polyhydramnios	Polyhydramnios
Routine pulse oximetry screening after birth	Negative	Positive	Positive	Positive	Negative	Negative	Negative	Negative	Negative
*Cardiovascular malformations by Echo*									
PVS (HP:0001642)	Positive	–	–	–	–	–	–	–	–
ASD (HP:0001684)	Positive	Positive	Positive	Positive	Positive	Positive	–	Positive	Positive
HCM (HP:0001639)	Positive	–	Positive	Positive	–	–	–	–	–
Other cardiac anomalies	Mitral valve defects (HP:0001633)	PDA (HP:0001643)	tricuspid valve defects (HP:0001702)	PDA (HP:0001643)、Mitral valve defects(HP:0001633)		VSD (HP:0001629)	PDA (HP:0001643)		
*Characteristic facies*									
Hypertelorism (HP:0000316)	–	–	–	Positive	–	–	–	–	–
Low-set ears (HP:0000369)	–	–	–	Positive	–	–	–	–	–
Other facial anomalies			High-arched palate (HP:0000218)、 Microphthalmia (HP:0000568)、Webbed neck (HP:0000465)			Sparse and curly hair (HP:0002212)、Long eyelashes (HP:0000527)			Micrognathia (HP:0000347)、Webbed neck (HP:0000465)
*Clinical features*									
Tachypnea (HP:0002790)	Positive	Positive	Positive	Positive	–	–	–	Positive	Positive
Cyanosis (HP:0000961)	Positive	Positive	–	Positive	–	–	–	–	
Feeding difficulties (HP:0008872)	–	Positive	–	Positive	–	Positive	–	–	Positive
Pleural effusion (HP:0002202)	–	–	–	Positive	–	–	–	–	Positive
Bleeding tendency (HP:0001892)	Positive	–	–	Positive	–	–	–	Positive	–
Cryptorchidism(HP:0000028)	–	Positive	–	Positive	–	–	–	–	–
Pectus excavatum (HP:0000767)	–	Positive	–	–	–	–	–	–	–
Other extracardiac anomalies		Abnormal renal pelvis morphology (HP:0010944)	Laryngomalacia (HP:0001601)、 Abnormal renal pelvis morphology (HP:0010944)	Abnormal renal pelvis morphology (HP:0010944)	Erythema (HP:0031286)		Arteriovenous malformation of the spine (HP:0002390)	Arteriovenous malformation of the brain (HP:108010)	Laryngomalacia (HP:0001601)、Abnormal renal pelvis morphology (HP:0010944)
Surgery or not	No	No	Yes	No	No	No	Yes	No	No
180 days of age outcome	ID at 20 weeks, 6 days	ID at 4 weeks, 3 days	Survival	ID at 10 weeks, 4 days	Survival	Survival	Survival	Survival	Survival

Abbreviations: ASD atrial septal defect, VSD ventricular septal defect, HCM hypertrophic cardiomyopathy, PDA patent ductus arteriosus, PVS pulmonary valve stenosis, ID infant death

### Cardiovascular manifestations

Cardiovascular abnormalities constituted the main clinical phenotype of RASopathies while the type of CHDs is different among the different disorders (Table 1). Secundum type atrial septal defect (ASD II) (HP: 0001684) was prominent in all individuals with NS and Noonan-related disorders (100%, n = 6/6). The ASD II was associated with other cardiac anomalies in different patients: concomitant mitral (HP: 0001633) or tricuspid (HP: 0001702) valve defects in P1, P3 and P4 (50%, 3/6), pulmonic valve stenosis (PVS) (HP: 0001642) in P1 and ventricular septal defect (VSD) (HP: 0001629) in P6. Three patients (P1, P3 and P4) developed hypertrophic cardiomyopathy (HCM) (HP: 0001639) during their neonatal period. Isolated ASD occurred in a male (P5) with NS. The CM-AVM-associated CHDs included patent ductus arteriosus (PDA) (HP: 0001643) which was isolated in P7 and associated with an ASD II in P8. Isolated ASD was identified in the patient with CS (P9).

### Other clinical features in RASopathies

**Fig. 1:**
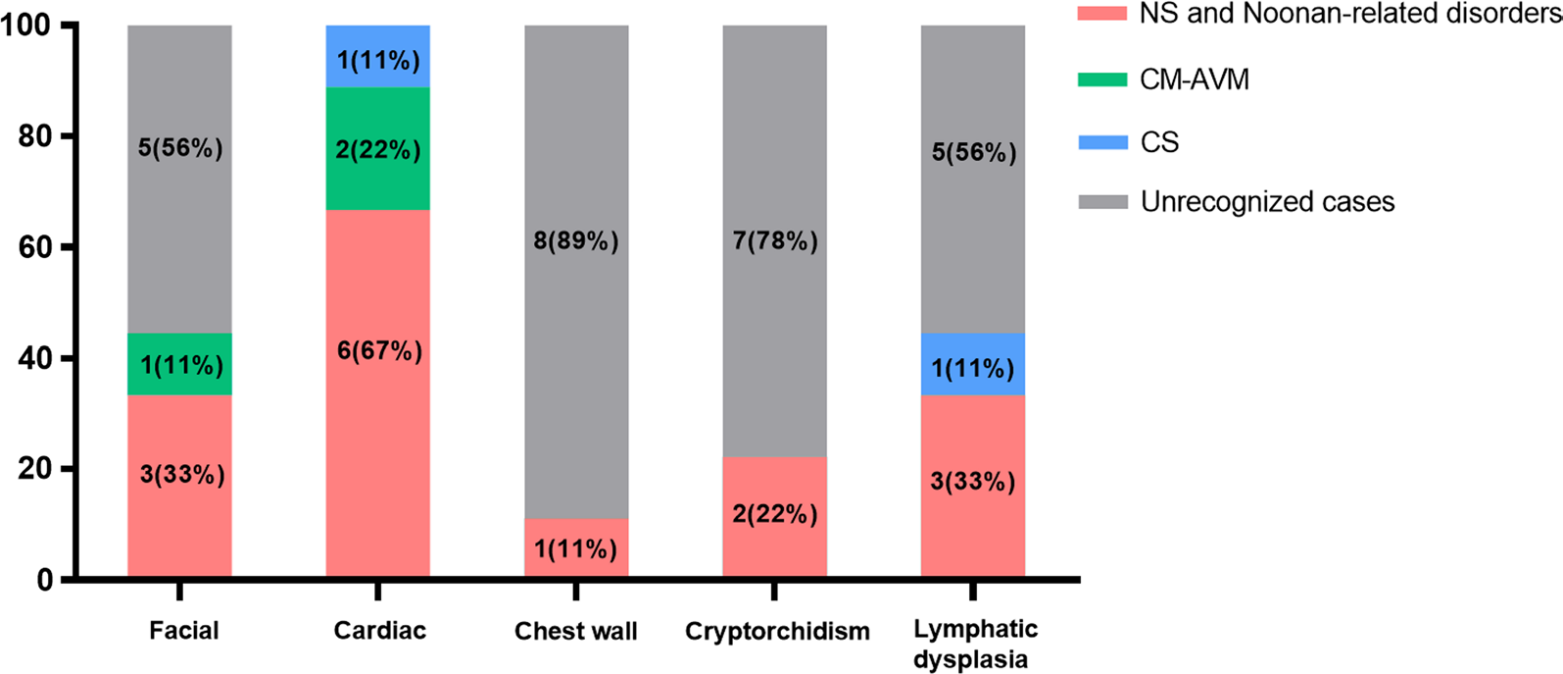
Distribution of major features in nine neonates with confirmed diagnosis of RASopathies in our cohort. As indicated in the legend, red, green and blue individually correspond to the number (percent) of the RASopathy categories and grey to those without the anomalies. Abbreviations: NS: Noonan syndrome; CM-AVM: capillary malformation-arteriovenous malformation; CS: Costello syndrome.

Since heterogeneity among neonates with RASopathies made early diagnosis with certainty difficult, we described their phenotypic spectrum, taking NS as the prototype, then focus on multiple extracardiac anomalies in addition to above CHDs that might distinguish the other syndromes (Fig. 1). Facial features identified in 2 (P3 and P4) out of 5 neonates with NS (40%, 2/5) included hypertelorism (HP: 0000316)/microphthalmia (HP: 0000568), high-arched palate (HP: 0000218) and low-set ears (HP: 0000369). The sparse curly hair (HP: 0002212) and long eyelashes (HP: 0000527) were observed in a female (P6) with NS-LAH. The patient (P9) with CS had a micrognathia (HP: 0000347) at birth. Other individuals (P1, P2, P5, P7 and P8) exhibited no distinctive facial features during the early neonatal period. Other extracardiac anomalies included abnormal renal pelvis morphology (HP: 0010944) in 3 patients (P2, P3 and P4) with NS and one (P9) with CS, cryptorchidism (HP: 0000028) in 2 patients (P2 and P4) with NS, laryngomalacia (HP: 0001601) in one (P3) with NS and another (P9) with CS, pleural effusion (HP: 0002202) in one (P4) with NS and another (P9) with CS, arteriovenous malformations (HP: 0100026) in 2 patients (P7 and P8) with CM-AVM, webbed neck (HP: 0000465) in one patient (P3) with NS and another (P9) with CS and pectus excavatum (HP: 0000767) in one patient (P2) with NS. Atypical skin rash (HP: 0000988) were observed in one patient (P5) with NS.

The major presentations for the initial visit to the NICUs in our cohort were summarized as followed. Six patients (P1, P2, P3, P4, P8 and P9) presented with tachypnea in the newborn period (range, 0–23 days) and three of them (P1, P2 and P4) had cyanosis concurrently that was attributable to their corresponding CHDs. Other common reasons were feeding difficulties in 3 patients (P2, P4 and P6) with NS and one (P9) with CS and bleeding tendency in 2 patients (P2 and P4) with NS and one (P8) with CM-AVM.

### Molecular analysis of 9 neonates with RASopathies

As shown in Table 2, a definitive molecular diagnosis for 9 patients was provided. All pathogenic variants were uploaded to Leiden Open Variation Database (LOVD) (http://databases.LOVD.nl/shared). In our study, we identified six genes harboring a total of 8 de novo variants. The *PTPN11*, *RAF1* and *RASA1* were identified in 2 probands each and the remaining 3 genes per each. Six patients with NS and Noonan-related disorders and one with CS had reported gene mutations, including *PTPN11* in 2 patients (P1 and P2), *RAF1* in 2 patients (P3 and P4), *BRAF* in 1 patient (P5), *SHOC2* in 1 patient (P6) and *HRAS* in 1 patient (P9).

**Table 2:** Molecular profiles of nine neonates with RASopathies

Patient number	LOVD Individual ID	Gene	Nucleotide change	Amino acid change	Molecular diagnosis	Inheritance pattern	Zygosity	Variant type	In silico prediction (SIFT/PolyPhen)	ACMG classification	Novel/ published (PMID)	De novo/ inherited	Age of confirmed diagnosis (d)
Patient 1	00410414	*PTPN11* (NM_002834.3)	c.1517A > C	p.Gln506Pro	Noonan syndrome 1 (MIM:163,950)	AD	Het	missense	–	Pathogenic (PS2, PM2, PP3, PP4)	Published (14961557)	De novo	61
Patient 2	00410466	*PTPN11* (NM_002834.3)	c.182A > G	p.Asp61Gly	Noonan syndrome 1 (MIM:163,950)	AD	Het	missense	–	Pathogenic (PS2, PM2, PP3, PP4)	Published (11704759)	De novo	49
Patient 3	00410416	*RAF1* (NM_002880.3)	c.770C > T	p.Ser257Leu	Noonan syndrome 5 (MIM:611,553)	AD	Het	missense	–	Pathogenic (PS2, PM2, PP3, PP4)	Published (17603482)	De novo	84
Patient 4	00410417	*RAF1* (NM_002880.3)	c.770C > T	p.Ser257Leu	Noonan syndrome 5 (MIM:611,553)	AD	Het	missense	–	Pathogenic (PS2, PM2, PP3, PP4)	Published (17603482)	De novo	78
Patient 5	00410430	*BRAF* (NM_004333.5)	c.1405G > A	p.Gly469Arg	Noonan syndrome 7 (MIM:613,706)	AD	Het	missense	–	Likely pathogenic (PS2, PM2, PP1,PP3, PP4)	Published (25157968)	De novo	58
Patient 6	00410431	*SHOC2* (NM_007373)	c.4A > G	p.Ser2Gly	Noonan syndrome -like with loose anagen hair 1 (MIM:607,721)	AD	Het	missense	–	Pathogenic (PS2, PM2, PP3, PP4)	Published (19684605)	De novo	50
Patient 7	00410432	*RASA1* (NM_002890.2)	*c.2828 T* > *C*	p.Leu943Pro	Capillary malformation- arteriovenous malformation 1 (MIM:608,354)	AD	Het	missense	Deleterious/ Probably damaging	Likely pathogenic (PS2, PM2, PM5, PP1, PP3, PP4)	Novel	De novo	64
Patient 8	00410433	*RASA1* (NM_002890.2)	*c.2001del*	p.Pro668Leufs*10	Capillary malformation- arteriovenous malformation 1 (MIM:608,354)	AD	Het	frameshift	–	Pathogenic (PVS1, PS2, PM2, PP3, PP4)	Novel	De novo	79
Patient 9	00410435	*HRAS* (NM_005343)	c.37G > T	p.Gly13Cys	Costello syndome (MIM:218,040)	AD	Het	missense	–	Pathogenic (PS2, PM2, PP3, PP4)	Published (16835863)	De novo	55

*Abbreviations* *AD* Autosomal recessive inheritance disease, *Het* Heterozygous, *WES* Whole-exome sequencing, *LOVD* Leiden open variation database, *ACMG* American college of medical genetics

*Variants were classified according to the 2015 ACMG/AMP standards

**Fig. 2:**
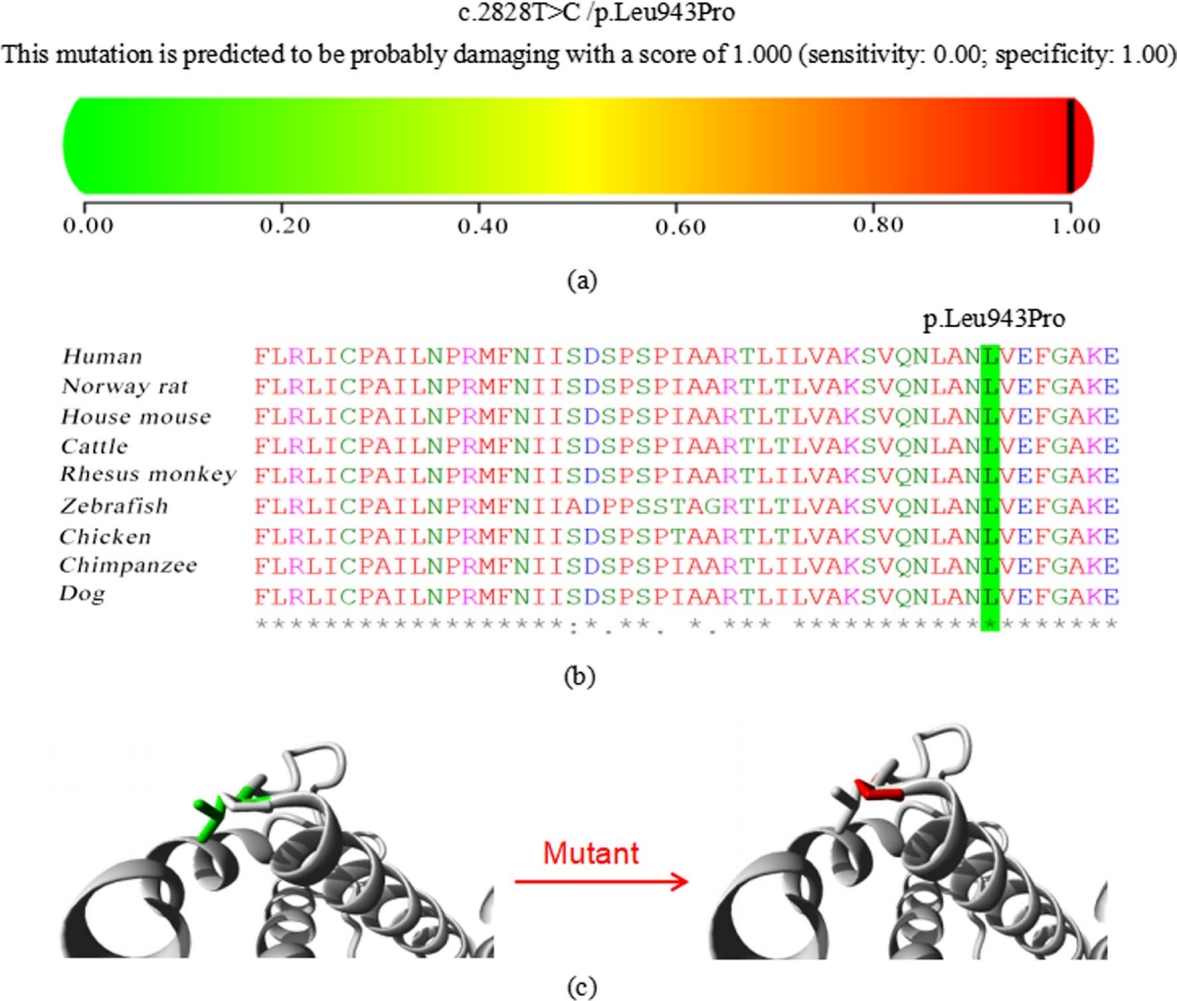
Detailed analysis of the novel missense variant (NM_002890.2: c.2828 T > C, p.Leu943Pro) in *RASA1* gene. **a** Pathogenicity analyses by PolyPhen-2. **b** In silico analysis in different species by T-Coffee Multiple Sequence Alignment Program. **c** 3D structure models of wild type and mutant type in *RASA1* by HOPE

In 2 patients (P7 and P8) with CM-AVM, novel variants in *RASA1* were detected. The novel missense variant, (NM_002890.2: c.2828 T > C, p.Leu943Pro), occurred with an amino acid change from a nonpolar amino acid of leucine (Leu) to another nonpolar amino acid of proline (Pro). It was found in a male (P7) presenting massive edema of the lower limbs (HP: 0010741) at birth and an initial chest X-ray demonstrated bilateral pleural effusions, pulmonary edema, and diffuse anasarca at admission. A bedside echocardiogram showed a cardiac overload. The neonate had magnetic resonance imaging (MRI) of his lower limbs on day of life (DOL) 9, which revealed extracranial arteriovenous fistulas (AVFs) (HP: 0,004,947) in spine. This variant was predicted to be “probably damaging” by PolyPhen-2 (Fig. 2a). The conservation analysis showed that the wild-type residue-Leu is conserved at this position (Fig. 2b). In addition, the RASA1 protein (the human RASA1 (UniProtKB/Swiss-Prot P20936) protein sequences were used as the reference sequence) was built based on a homologous structure using HOPE. The structure (Fig. 2c) revealed that the mutated residue-pro disrupted a α-helix leading to severe effects on the structure of the protein. ES identified another de novo in-frame deletion novel variant in *RASA1* gene (NM_002890.2: c.2001del, p.Pro668Leufs*10) where no pathogenic variants have been previously reported to be responsible for CM-AVM. Since the typical phenotypes of both patients appropriately matched the disorder, 2 novel variants were considered to be disease-causing.

### 180-Day outcomes in 9 neonates with RASopathies

During a 180-day of age follow-up interval, all patients in our cohort survived the neonatal period. At the end of the 6 months of age, 3 patients (P1, P2 and P4) diagnosed with NS died (Table 1). The 180-day mortality rate was 33.33% (3/9).

For the survival and severe manifestations/complications of RASopathies, we found among the 3 patients (P1, P2 and P4) who died, 2 patients (P1 and P4) with NS suffered severe coagulation defects and electrolyte disturbances which were difficult to correct during their hospital stay. HCM coexisted with structural malformations in both, evolving to congestive heart failure which may explain their worse survival. Another patient (P2) with NS presented persistent tachypnea and cyanosis because of the underlying heart failure from birth and developed feeding difficulties during early neonatal period. These life-threatening conditions made their families to give up treatments and discharge automatically.

We then evaluated the impact of expeditious treatments on the survival of this cohort. During follow-up, the clinical course of a patient (P3) with NS showed progressive aggravation of right ventricular hypertrophy compared with the initial echocardiographic evaluation. ASD device closure was performed at the age of 35 days after birth. A male (P7) with CM-AVM had life-threatening congestive heart failure from birth. The percutaneous cardiac catheterization and imaging were performed. A macrofistulas in spine was identified. This male patient was surgically treated after counseling with a multidisciplinary team including specialists in interventional radiology, surgery and cardiology. Both patients were in clinical remission until now.

## Discussion

CHD is a large, rapidly emerging global problem in child health [19]. Without the ability to substantially alter the prevalence of CHD, early diagnoses and interventions must be used to improve survival. In this study, we presented a detailed clinical course of 9 neonates presenting various CHDs and having a confirmed genetic diagnosis of RASopathies due to de novo pathogenic variants. Our results emphasized the importance of family-based ES in neonates presenting with critical cardiac lesions, multiple extracardiac anomalies and other atypical symptoms with a suspected genetic etiology.

Cardiac symptoms were the most common initial presentation in children with RASopathies [20–22]. In this study, all individuals have cardiovascular abnormalities by initial echocardiographic evaluation. Previous studies indicated that the type of cardiac involvement was different among the different disorders [23, 24]. In our cohort, ASDII were detected in all individuals (P1, P2, P3, P4, P5 and P6) with NS and Noonan-related disorders. Three of them (P1, P3 and P4) developed HCM. A female with CS (P9) had isolated atrial septal defect. Furthermore, we found that the characteristic multiple extracardiac anomalies [7] were less noticeable during neonatal period and only two diagnose (P3 and P4) was included in the diagnostic criteria for NS proposed by van der Burgt [25]. Our results indicated that characteristic facies (P1, P2 and P5), typical chest wall (P1, P3, P4 and P5) and short stature (P1, P2, P3, P4 and P5) were clinically insignificant in most patients with NS. In spite of the unrecognized phenotypes in neonatal period, some reports have been made on the prenatal ultrasound findings in RASopathies [26, 27]. We detected four affected pregnancies (P2, P3, P8 and P9) in which prenatal ultrasonography demonstrated fetal abnormalities, providing clues for a suspected genetic etiology. Another five cases (P1, P4, P5, P6 and P7) had a normal ultrasound scan prenatally. This meant that prenatal diagnosis of RASopathies cannot be easily performed because only few of the phenotype features can be detected by ultrasound.

The germline pathogenic variants affect RAS-mitogen activated protein kinase (MAPK) pathway genes, resulting in comparable clinical phenotypes [28]. Nine patients in this study were sporadic cases and all harbored dominant de novo variants. Although trio-based Exome and genome sequencing have greatly facilitated the detection of DNMs in rare genetic disease, the current knowledge of its biogenesis mechanisms and functional effects remains limited. One of the prominent risk factors concerning germline de novo variants is the fact that their number increases steadily with the age of the father at conception. Our study cohort revealed that paternal ages at conception ranged from 28 to 43 y/o (median: 34.5 y/o), indicating mostly older fathers. Some studies were published in individuals with CHDs and also documented a paternal age effect on germline mutation rates in individuals with CHDs [4, 29, 30]. Collectively, these studies demonstrated that the dominant de novo variant rate among individuals might partially due to paternal age. With respect to the detection of novel de novo variants, interpreting their pathogenicity becomes more important. Our study assessed two novel de novo variants in two patients (P7 and P8) with CM-AVM and the results highlighted the relevance of combining bioinformatics analyses and clinical correlations for confidence that a particular de novo variant was disease-causing. For the frameshift one in P8 (NM_002890.2: c.2001del, p.Pro668Leufs*10), this type of mutation likely results in deleterious consequences. For the missense one in P7 (NM_002890.2: c.2828 T > C, p.Leu943Pro), we evaluated the evolutionary conservation of the affected nucleotide by T-Coffee, scored its functional impact using PolyPhen-2 and performed protein structure analysis by HOPE. Evidence of causal roles of two variant were additionally provided by their clinical correlations.

As with other rare diseases, the sole option for children with RASopathies has long been symptomatic therapies, notably for cardiopathies and growth delay [20–22, 31]. In this study, for both patients (P3 and P7) evolving to serious heart failure, beneficial effects of ASD device closure and intervention for AVFs in spine have been observed during our follow-up till 180-days of age. However, three patients with NS progressively aggravated and 2 of them (P1 and P4) had severe coagulation impairments and congestive heart failure caused by HCM. Another one (P2) required catheterization and had feeding difficulties. These might partially contribute to the clinical decision-making process and worse survival after the establishment of a molecular diagnosis.

Finally, our results demonstrated again that the power of family-based NGS was indisputable in the immediate detection of disorders that are clinically atypical and unrecognized in critically ill infants with CHDs. In a recent study [4], the trio-ES as first-line test in CHD probands revealed that DNMs accounted for 8% of cases. It is deemed that DNMs are more deleterious, on average, than inherited variation because they have been subjected to less stringent evolutionary selection [32, 33]. Consequently, an open question raised about the probable risks to existing or future siblings of the affected proband when parents who have had a child with a genetic disease caused by a DNM. Although the recurrence risk would be negligible if DNMs occurred exclusively in germ cells, new mutations can and do occur at any stage of gametogenesis and, indeed, of development, leading the indispensability of genetic counselling for DNMs [34]. We hope to find these answers in research yet to set up.

## Conclusion

Our results highlighted the inherent limitations in phenotype-driven genetic testing in the neonatal population and support the application of family-based ES as a first-tier test in neonates with CHDs and a suspected genetic etiology.

## Data Availability

The datasets generated and/or analysed during the current study are available in the Leiden Open Variation Database (LOVD) repository, [http://databases.LOVD.nl/shared/].

## Abbreviations

CHDs: Congenital heart defects
DNMs: De novo mutations
ES: Exome sequencing
NGS: Next-generation-sequencing
NS: Noonan syndrome
NSML: Noonan syndrome with multiple lentigines
NS-LAH: Noonan-like syndrome with loose anagen hair
CFC: Cardio-facio-cutaneous syndrome
CS: Costello syndrome
CM-AVM: Capillary malformation-arteriovenous malformation
XH: Xinhua Hospital affiliated with Shanghai Jiao Tong University School of Medicine
CH: Children's Hospital affiliated with Shanghai Jiao Tong University
NICUs: Neonatal intensive care units
EMRs: Electronic medical records
HPO: Human phenotype ontology
CNVs: Copy number variations
CMA: Chromosomal microarray analysis
ASD II: Secundum type atrial septal defect
VSD: Ventricular septal defect
HCM: Hypertrophic cardiomyopathy
PDA: Patent ductus arteriosus
PVS: Pulmonary valve stenosis
MRI: Magnetic resonance imaging
DOL: Day of life
AVFs: Arteriovenous fistulas
MAPK: RAS-mitogen activated protein kinase
